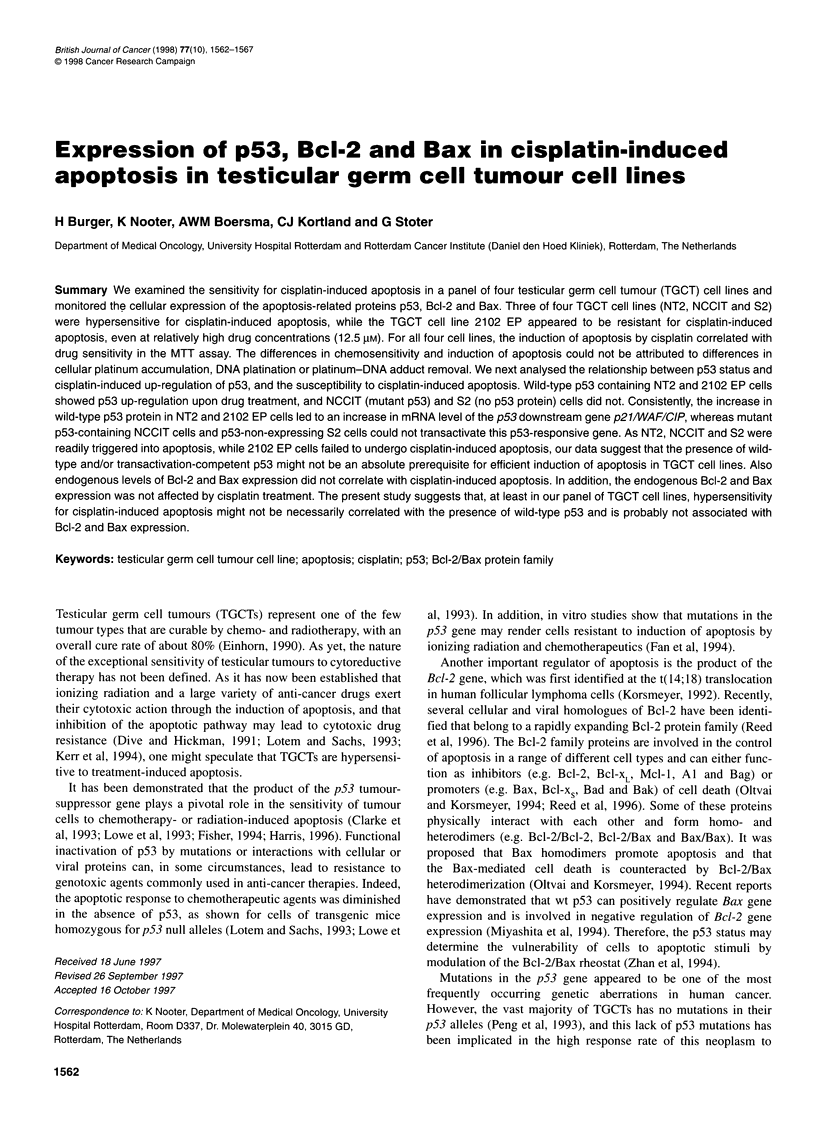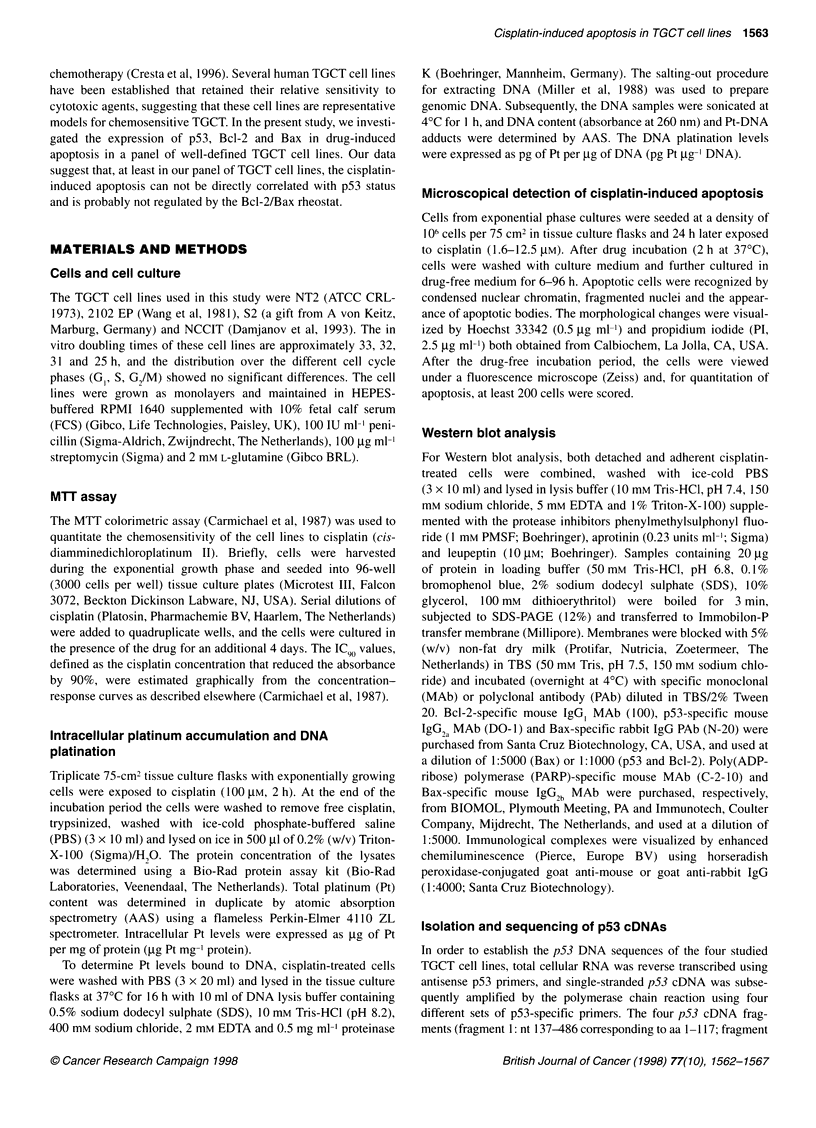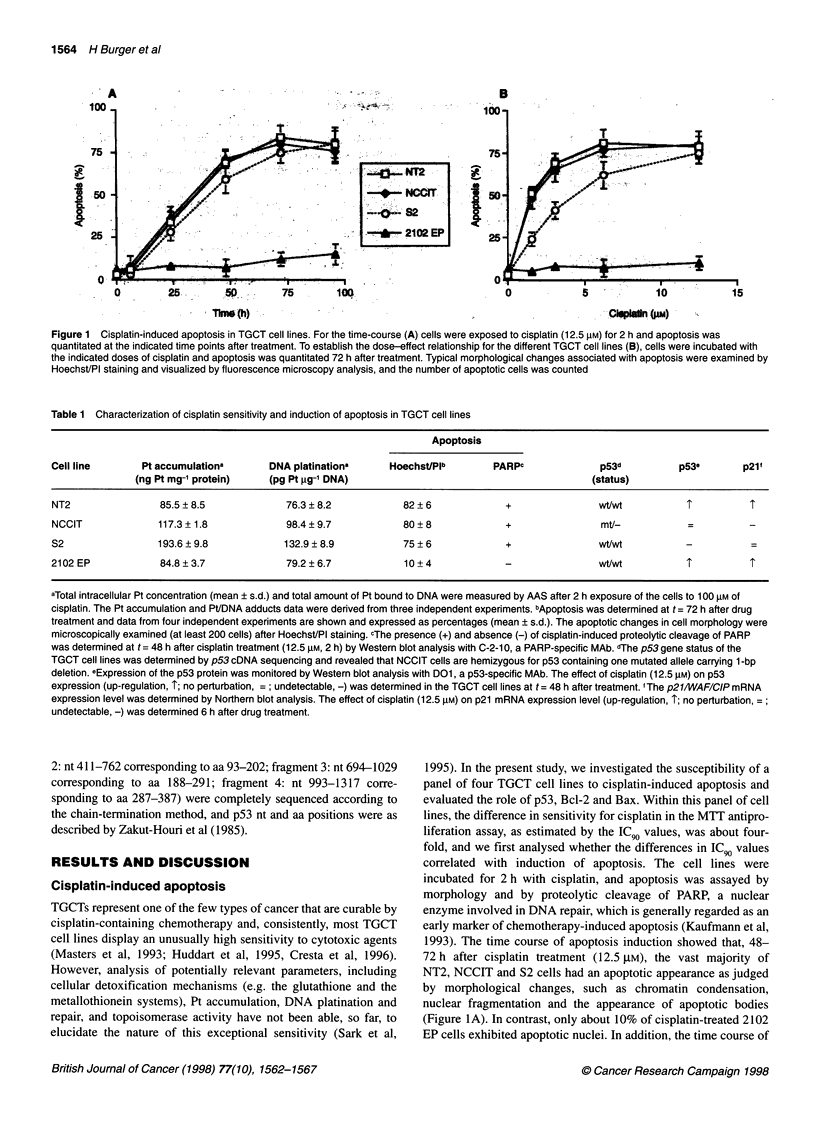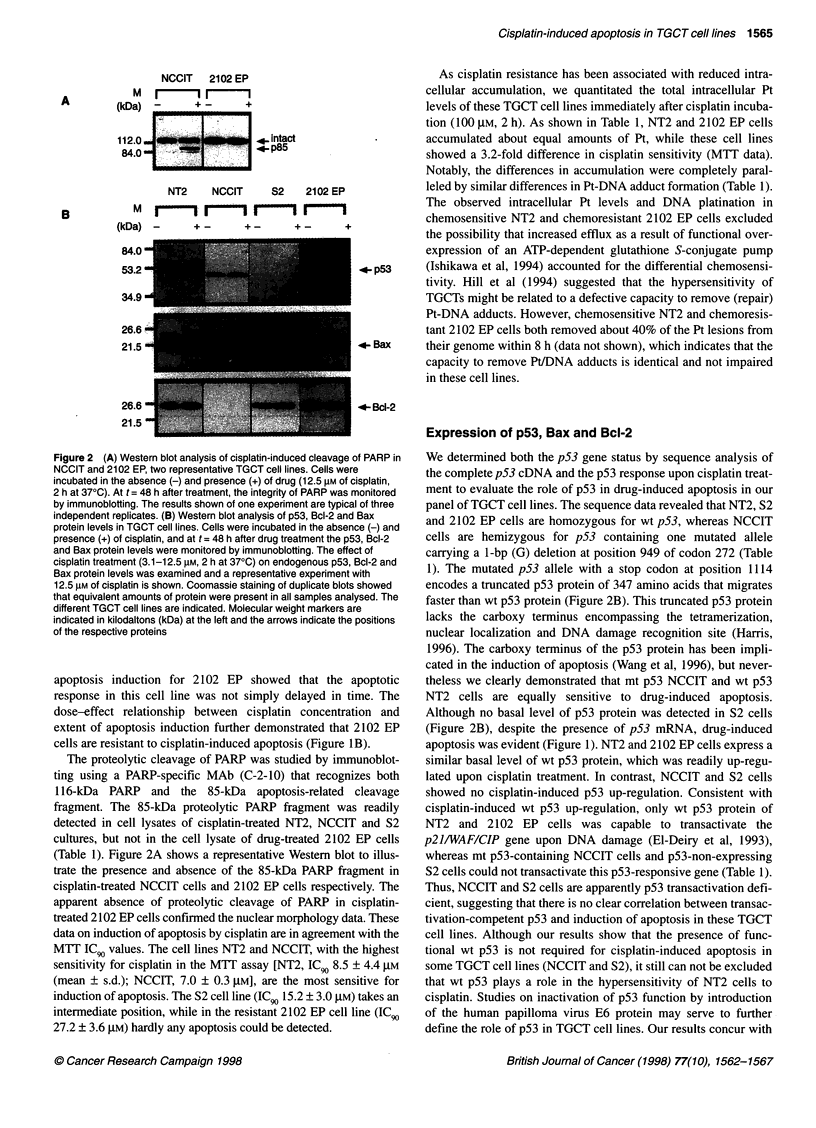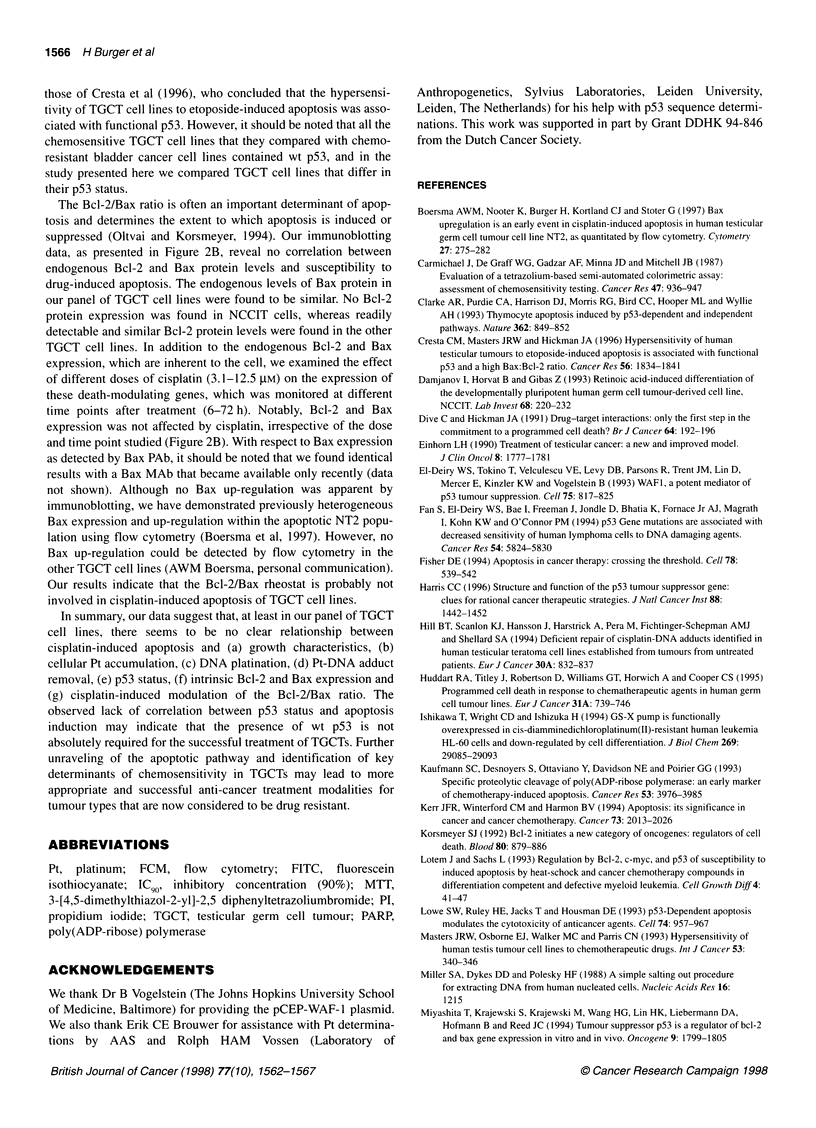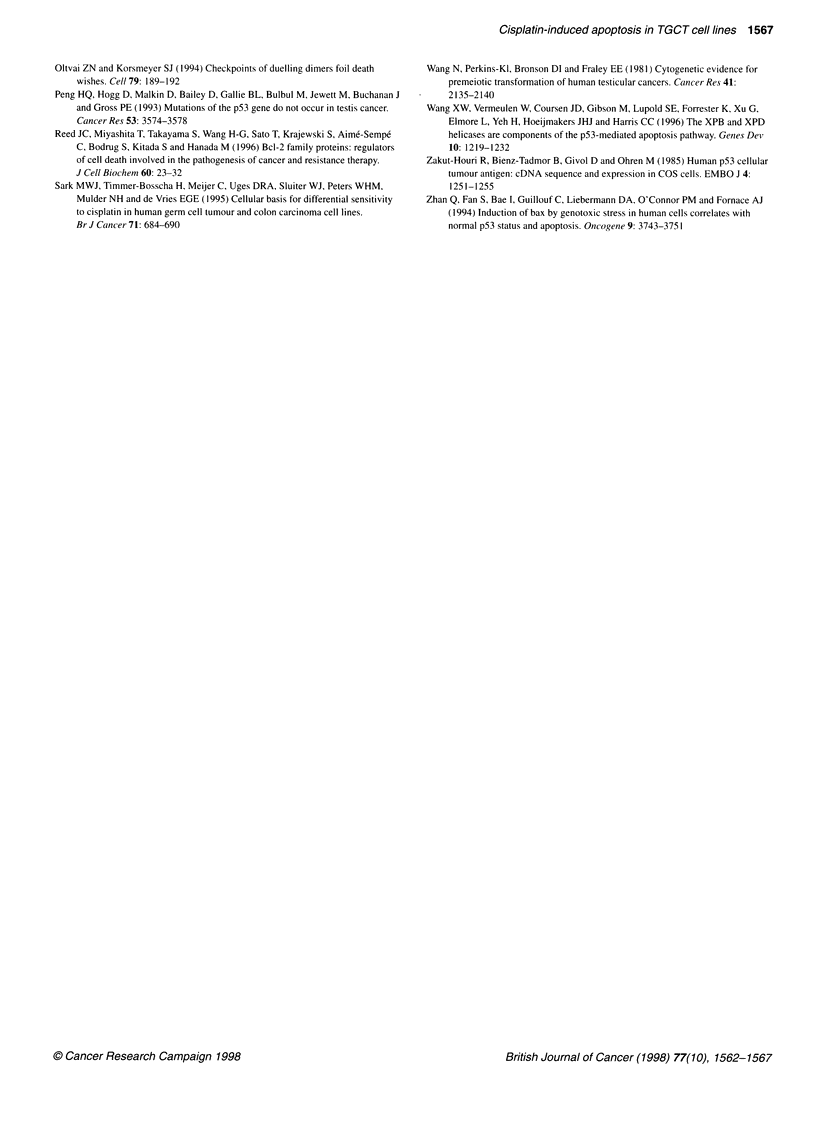# Expression of p53, Bcl-2 and Bax in cisplatin-induced apoptosis in testicular germ cell tumour cell lines.

**DOI:** 10.1038/bjc.1998.257

**Published:** 1998-05

**Authors:** H. Burger, K. Nooter, A. W. Boersma, C. J. Kortland, G. Stoter

**Affiliations:** Department of Medical Oncology, University Hospital Rotterdam and Rotterdam Cancer Institute (Daniel den Hoed Kliniek), The Netherlands.

## Abstract

**Images:**


British Journal of Cancer (1998) 77(10), 1562–1567
© 1998 Cancer Research Campaign

### Expression of p53, Bcl-2 and Bax in cisplatin-induced apoptosis in testicular germ cell tumour cell lines

**H Burger, K Nooter, AWM Boersma, CJ Kortland and G Stoter**

Department of Medical Oncology, University Hospital Rotterdam and Rotterdam Cancer Institute (Daniel den Hoed Kliniek), Rotterdam, The Netherlands

**Summary** We examined the sensitivity for cisplatin-induced apoptosis in a panel of four testicular germ cell tumour (TGCT) cell lines and monitored the cellular expression of the apoptosis-related proteins p53, Bcl-2 and Bax. Three of four TGCT cell lines (NT2, NCCIT and S2) were hypersensitive for cisplatin-induced apoptosis, while the TGCT cell line 2102 EP appeared to be resistant for cisplatin-induced apoptosis, even at relatively high drug concentrations (12.5 μM). For all four cell lines, the induction of apoptosis by cisplatin correlated with drug sensitivity in the MTT assay. The differences in chemosensitivity and induction of apoptosis could not be attributed to differences in cellular platinum accumulation, DNA platination or platinum–DNA adduct removal. We next analysed the relationship between p53 status and cisplatin-induced up-regulation of p53, and the susceptibility to cisplatin-induced apoptosis. Wild-type p53 containing NT2 and 2102 EP cells showed p53 up-regulation upon drug treatment, and NCCIT (mutant p53) and S2 (no p53 protein) cells did not. Consistently, the increase in wild-type p53 protein in NT2 and 2102 EP cells led to an increase in mRNA level of the *p53* downstream gene *p21/WAF/CIP*, whereas mutant p53-containing NCCIT cells and p53-non-expressing S2 cells could not transactivate this p53-responsive gene. As NT2, NCCIT and S2 were readily triggered into apoptosis, while 2102 EP cells failed to undergo cisplatin-induced apoptosis, our data suggest that the presence of wild-type and/or transactivation-competent p53 might not be an absolute prerequisite for efficient induction of apoptosis in TGCT cell lines. Also endogenous levels of Bcl-2 and Bax expression did not correlate with cisplatin-induced apoptosis. In addition, the endogenous Bcl-2 and Bax expression was not affected by cisplatin treatment. The present study suggests that, at least in our panel of TGCT cell lines, hypersensitivity for cisplatin-induced apoptosis might not be necessarily correlated with the presence of wild-type p53 and is probably not associated with Bcl-2 and Bax expression.

**Keywords:** testicular germ cell tumour cell line; apoptosis; cisplatin; p53; Bcl-2/Bax protein family

Testicular germ cell tumours (TGCTs) represent one of the few tumour types that are curable by chemo- and radiotherapy, with an overall cure rate of about 80% (Einhorn, 1990). As yet, the nature of the exceptional sensitivity of testicular tumours to cytoreductive therapy has not been defined. As it has now been established that ionizing radiation and a large variety of anti-cancer drugs exert their cytotoxic action through the induction of apoptosis, and that inhibition of the apoptotic pathway may lead to cytotoxic drug resistance (Dive and Hickman, 1991; Lotem and Sachs, 1993; Kerr et al, 1994), one might speculate that TGCTs are hypersensitive to treatment-induced apoptosis.

It has been demonstrated that the product of the *p53* tumour-suppressor gene plays a pivotal role in the sensitivity of tumour cells to chemotherapy- or radiation-induced apoptosis (Clarke et al, 1993; Lowe et al, 1993; Fisher, 1994; Harris, 1996). Functional inactivation of p53 by mutations or interactions with cellular or viral proteins can, in some circumstances, lead to resistance to genotoxic agents commonly used in anti-cancer therapies. Indeed, the apoptotic response to chemotherapeutic agents was diminished in the absence of p53, as shown for cells of transgenic mice homozygous for *p53* null alleles (Lotem and Sachs, 1993; Lowe et al, 1993). In addition, in vitro studies show that mutations in the *p53* gene may render cells resistant to induction of apoptosis by ionizing radiation and chemotherapeutics (Fan et al, 1994).

*Received 18 June 1997*
*Revised 26 September 1997*
*Accepted 16 October 1997*

*Correspondence to:* K Nooter, Department of Medical Oncology, University Hospital Rotterdam, Room D337, Dr. Molewaterplein 40, 3015 GD, Rotterdam, The Netherlands

Another important regulator of apoptosis is the product of the *Bcl-2* gene, which was first identified at the t(14;18) translocation in human follicular lymphoma cells (Korsmeyer, 1992). Recently, several cellular and viral homologues of Bcl-2 have been identified that belong to a rapidly expanding Bcl-2 protein family (Reed et al, 1996). The Bcl-2 family proteins are involved in the control of apoptosis in a range of different cell types and can either function as inhibitors (e.g. Bcl-2, Bcl-$x_L$, Mcl-1, A1 and Bag) or promoters (e.g. Bax, Bcl-$x_S$, Bad and Bak) of cell death (Oltvai and Korsmeyer, 1994; Reed et al, 1996). Some of these proteins physically interact with each other and form homo- and heterodimers (e.g. Bcl-2/Bcl-2, Bcl-2/Bax and Bax/Bax). It was proposed that Bax homodimers promote apoptosis and that the Bax-mediated cell death is counteracted by Bcl-2/Bax heterodimerization (Oltvai and Korsmeyer, 1994). Recent reports have demonstrated that wt p53 can positively regulate *Bax* gene expression and is involved in negative regulation of *Bcl-2* gene expression (Miyashita et al, 1994). Therefore, the p53 status may determine the vulnerability of cells to apoptotic stimuli by modulation of the Bcl-2/Bax rheostat (Zhan et al, 1994).

Mutations in the *p53* gene appeared to be one of the most frequently occurring genetic aberrations in human cancer. However, the vast majority of TGCTs has no mutations in their *p53* alleles (Peng et al, 1993), and this lack of p53 mutations has been implicated in the high response rate of this neoplasm to chemotherapy (Cresta et al, 1996). Several human TGCT cell lines have been established that retained their relative sensitivity to cytotoxic agents, suggesting that these cell lines are representative models for chemosensitive TGCT. In the present study, we investigated the expression of p53, Bcl-2 and Bax in drug-induced apoptosis in a panel of well-defined TGCT cell lines. Our data suggest that, at least in our panel of TGCT cell lines, the cisplatin-induced apoptosis can not be directly correlated with p53 status and is probably not regulated by the Bcl-2/Bax rheostat.

### MATERIALS AND METHODS

### Cells and cell culture

The TGCT cell lines used in this study were NT2 (ATCC CRL-1973), 2102 EP (Wang et al, 1981), S2 (a gift from A von Keitz, Marburg, Germany) and NCCIT (Damjanov et al, 1993). The in vitro doubling times of these cell lines are approximately 33, 32, 31 and 25 h, and the distribution over the different cell cycle phases ($G_1$, S, $G_2$/M) showed no significant differences. The cell lines were grown as monolayers and maintained in HEPES-buffered RPMI 1640 supplemented with 10% fetal calf serum (FCS) (Gibco, Life Technologies, Paisley, UK), 100 IU $ml^{-1}$ penicillin (Sigma-Aldrich, Zwijndrecht, The Netherlands), 100 μg $ml^{-1}$ streptomycin (Sigma) and 2 mM L-glutamine (Gibco BRL).

### MTT assay

The MTT colorimetric assay (Carmichael et al, 1987) was used to quantitate the chemosensitivity of the cell lines to cisplatin (*cis*-diamminedichloroplatinum II). Briefly, cells were harvested during the exponential growth phase and seeded into 96-well (3000 cells per well) tissue culture plates (Microtest III, Falcon 3072, Beckton Dickinson Labware, NJ, USA). Serial dilutions of cisplatin (Platosin, Pharmachemie BV, Haarlem, The Netherlands) were added to quadruplicate wells, and the cells were cultured in the presence of the drug for an additional 4 days. The $IC_{90}$ values, defined as the cisplatin concentration that reduced the absorbance by 90%, were estimated graphically from the concentration–response curves as described elsewhere (Carmichael et al, 1987).

### Intracellular platinum accumulation and DNA platination

Triplicate 75-$cm^2$ tissue culture flasks with exponentially growing cells were exposed to cisplatin (100 μM, 2 h). At the end of the incubation period the cells were washed to remove free cisplatin, trypsinized, washed with ice-cold phosphate-buffered saline (PBS) (3 × 10 ml) and lysed on ice in 500 μl of 0.2% (w/v) Triton-X-100 (Sigma)/$H_2O$. The protein concentration of the lysates was determined using a Bio-Rad protein assay kit (Bio-Rad Laboratories, Veenendaal, The Netherlands). Total platinum (Pt) content was determined in duplicate by atomic absorption spectrometry (AAS) using a flameless Perkin-Elmer 4110 ZL spectrometer. Intracellular Pt levels were expressed as μg of Pt per mg of protein (μg Pt $mg^{-1}$ protein).

To determine Pt levels bound to DNA, cisplatin-treated cells were washed with PBS (3 × 20 ml) and lysed in the tissue culture flasks at 37°C for 16 h with 10 ml of DNA lysis buffer containing 0.5% sodium dodecyl sulphate (SDS), 10 mM Tris-HCl (pH 8.2), 400 mM sodium chloride, 2 mM EDTA and 0.5 mg $ml^{-1}$ proteinase K (Boehringer, Mannheim, Germany). The salting-out procedure for extracting DNA (Miller et al, 1988) was used to prepare genomic DNA. Subsequently, the DNA samples were sonicated at 4°C for 1 h, and DNA content (absorbance at 260 nm) and Pt-DNA adducts were determined by AAS. The DNA platination levels were expressed as pg of Pt per μg of DNA (pg Pt $μg^{-1}$ DNA).

### Microscopical detection of cisplatin-induced apoptosis

Cells from exponential phase cultures were seeded at a density of $10^6$ cells per 75 $cm^2$ in tissue culture flasks and 24 h later exposed to cisplatin (1.6–12.5 μM). After drug incubation (2 h at 37°C), cells were washed with culture medium and further cultured in drug-free medium for 6–96 h. Apoptotic cells were recognized by condensed nuclear chromatin, fragmented nuclei and the appearance of apoptotic bodies. The morphological changes were visualized by Hoechst 33342 (0.5 μg $ml^{-1}$) and propidium iodide (PI, 2.5 μg $ml^{-1}$) both obtained from Calbiochem, La Jolla, CA, USA. After the drug-free incubation period, the cells were viewed under a fluorescence microscope (Zeiss) and, for quantitation of apoptosis, at least 200 cells were scored.

### Western blot analysis

For Western blot analysis, both detached and adherent cisplatin-treated cells were combined, washed with ice-cold PBS (3 × 10 ml) and lysed in lysis buffer (10 mM Tris-HCl, pH 7.4, 150 mM sodium chloride, 5 mM EDTA and 1% Triton-X-100) supplemented with the protease inhibitors phenylmethylsulphonyl fluoride (1 mM PMSF; Boehringer), aprotinin (0.23 units $ml^{-1}$; Sigma) and leupeptin (10 μM; Boehringer). Samples containing 20 μg of protein in loading buffer (50 mM Tris-HCl, pH 6.8, 0.1% bromophenol blue, 2% sodium dodecyl sulphate (SDS), 10% glycerol, 100 mM dithioerythritol) were boiled for 3 min, subjected to SDS-PAGE (12%) and transferred to Immobilon-P transfer membrane (Millipore). Membranes were blocked with 5% (w/v) non-fat dry milk (Protifar, Nutricia, Zoetermeer, The Netherlands) in TBS (50 mM Tris, pH 7.5, 150 mM sodium chloride) and incubated (overnight at 4°C) with specific monoclonal (MAb) or polyclonal antibody (PAb) diluted in TBS/2% Tween 20. Bcl-2-specific mouse $IgG_1$ MAb (100), p53-specific mouse $IgG_{2a}$ MAb (DO-1) and Bax-specific rabbit IgG PAb (N-20) were purchased from Santa Cruz Biotechnology, CA, USA, and used at a dilution of 1:5000 (Bax) or 1:1000 (p53 and Bcl-2). Poly(ADP-ribose) polymerase (PARP)-specific mouse MAb (C-2-10) and Bax-specific mouse $IgG_{2b}$ MAb were purchased, respectively, from BIOMOL, Plymouth Meeting, PA and Immunotech, Coulter Company, Mijdrecht, The Netherlands, and used at a dilution of 1:5000. Immunological complexes were visualized by enhanced chemiluminescence (Pierce, Europe BV) using horseradish peroxidase-conjugated goat anti-mouse or goat anti-rabbit IgG (1:4000; Santa Cruz Biotechnology).

### Isolation and sequencing of p53 cDNAs

In order to establish the *p53* DNA sequences of the four studied TGCT cell lines, total cellular RNA was reverse transcribed using antisense p53 primers, and single-stranded *p53* cDNA was subsequently amplified by the polymerase chain reaction using four different sets of p53-specific primers. The four *p53* cDNA fragments (fragment 1: nt 137–486 corresponding to aa 1–117; fragment

© Cancer Research Campaign 1998

**Figure 1** Cisplatin-induced apoptosis in TGCT cell lines. For the time-course (**A**) cells were exposed to cisplatin (12.5 μM) for 2 h and apoptosis was quantitated at the indicated time points after treatment. To establish the dose–effect relationship for the different TGCT cell lines (**B**), cells were incubated with the indicated doses of cisplatin and apoptosis was quantitated 72 h after treatment. Typical morphological changes associated with apoptosis were examined by Hoechst/PI staining and visualized by fluorescence microscopy analysis, and the number of apoptotic cells was counted

**Table 1** Characterization of cisplatin sensitivity and induction of apoptosis in TGCT cell lines

| Cell line | Pt accumulation[a] (ng Pt $mg^{-1}$ protein) | DNA platination[a] (pg Pt $\mu g^{-1}$ DNA) | Apoptosis Hoechst/PI[b] | Apoptosis PARP[c] | p53[d] (status) | p53[e] | p21[f] |
|---|---|---|---|---|---|---|---|
| NT2 | 85.5 ± 8.5 | 76.3 ± 8.2 | 82 ± 6 | + | wt/wt | ↑ | ↑ |
| NCCIT | 117.3 ± 1.8 | 98.4 ± 9.7 | 80 ± 8 | + | mt/– | = | – |
| S2 | 193.6 ± 9.8 | 132.9 ± 8.9 | 75 ± 6 | + | wt/wt | – | = |
| 2102 EP | 84.8 ± 3.7 | 79.2 ± 6.7 | 10 ± 4 | – | wt/wt | ↑ | ↑ |

[a]Total intracellular Pt concentration (mean ± s.d.) and total amount of Pt bound to DNA were measured by AAS after 2 h exposure of the cells to 100 μM of cisplatin. The Pt accumulation and Pt/DNA adducts data were derived from three independent experiments. [b]Apoptosis was determined at $t = 72$ h after drug treatment and data from four independent experiments are shown and expressed as percentages (mean ± s.d.). The apoptotic changes in cell morphology were microscopically examined (at least 200 cells) after Hoechst/PI staining. [c]The presence (+) and absence (–) of cisplatin-induced proteolytic cleavage of PARP was determined at $t = 48$ h after cisplatin treatment (12.5 μM, 2 h) by Western blot analysis with C-2-10, a PARP-specific MAb. [d]The *p53* gene status of the TGCT cell lines was determined by *p53* cDNA sequencing and revealed that NCCIT cells are hemizygous for p53 containing one mutated allele carrying 1-bp deletion. [e]Expression of the p53 protein was monitored by Western blot analysis with DO1, a p53-specific MAb. The effect of cisplatin (12.5 μM) on p53 expression (up-regulation, ↑; no perturbation, = ; undetectable, –) was determined in the TGCT cell lines at $t = 48$ h after treatment. [f]The *p21/WAF/CIP* mRNA expression level was determined by Northern blot analysis. The effect of cisplatin (12.5 μM) on p21 mRNA expression level (up-regulation, ↑; no perturbation, = ; undetectable, –) was determined 6 h after drug treatment.

2: nt 411–762 corresponding to aa 93–202; fragment 3: nt 694–1029 corresponding to aa 188–291; fragment 4: nt 993–1317 corresponding to aa 287–387) were completely sequenced according to the chain-termination method, and p53 nt and aa positions were as described by Zakut-Houri et al (1985).

### RESULTS AND DISCUSSION

### Cisplatin-induced apoptosis

TGCTs represent one of the few types of cancer that are curable by cisplatin-containing chemotherapy and, consistently, most TGCT cell lines display an unusually high sensitivity to cytotoxic agents (Masters et al, 1993; Huddart et al, 1995, Cresta et al, 1996). However, analysis of potentially relevant parameters, including cellular detoxification mechanisms (e.g. the glutathione and the metallothionein systems), Pt accumulation, DNA platination and repair, and topoisomerase activity have not been able, so far, to elucidate the nature of this exceptional sensitivity (Sark et al, 1995). In the present study, we investigated the susceptibility of a panel of four TGCT cell lines to cisplatin-induced apoptosis and evaluated the role of p53, Bcl-2 and Bax. Within this panel of cell lines, the difference in sensitivity for cisplatin in the MTT antiproliferation assay, as estimated by the $IC_{90}$ values, was about four-fold, and we first analysed whether the differences in $IC_{90}$ values correlated with induction of apoptosis. The cell lines were incubated for 2 h with cisplatin, and apoptosis was assayed by morphology and by proteolytic cleavage of PARP, a nuclear enzyme involved in DNA repair, which is generally regarded as an early marker of chemotherapy-induced apoptosis (Kaufmann et al, 1993). The time course of apoptosis induction showed that, 48–72 h after cisplatin treatment (12.5 μM), the vast majority of NT2, NCCIT and S2 cells had an apoptotic appearance as judged by morphological changes, such as chromatin condensation, nuclear fragmentation and the appearance of apoptotic bodies (Figure 1A). In contrast, only about 10% of cisplatin-treated 2102 EP cells exhibited apoptotic nuclei. In addition, the time course of

 *© Cancer Research Campaign 1998*

**Figure 2** (**A**) Western blot analysis of cisplatin-induced cleavage of PARP in NCCIT and 2102 EP, two representative TGCT cell lines. Cells were incubated in the absence (–) and presence (+) of drug (12.5 μM of cisplatin, 2 h at 37°C). At $t$ = 48 h after treatment, the integrity of PARP was monitored by immunoblotting. The results shown of one experiment are typical of three independent replicates. (**B**) Western blot analysis of p53, Bcl-2 and Bax protein levels in TGCT cell lines. Cells were incubated in the absence (–) and presence (+) of cisplatin, and at $t$ = 48 h after drug treatment the p53, Bcl-2 and Bax protein levels were monitored by immunoblotting. The effect of cisplatin treatment (3.1–12.5 μM, 2 h at 37°C) on endogenous p53, Bcl-2 and Bax protein levels was examined and a representative experiment with 12.5 μM of cisplatin is shown. Coomassie staining of duplicate blots showed that equivalent amounts of protein were present in all samples analysed. The different TGCT cell lines are indicated. Molecular weight markers are indicated in kilodaltons (kDa) at the left and the arrows indicate the positions of the respective proteins apoptosis induction for 2102 EP showed that the apoptotic response in this cell line was not simply delayed in time. The dose–effect relationship between cisplatin concentration and extent of apoptosis induction further demonstrated that 2102 EP cells are resistant to cisplatin-induced apoptosis (Figure 1B).

The proteolytic cleavage of PARP was studied by immunoblotting using a PARP-specific MAb (C-2-10) that recognizes both 116-kDa PARP and the 85-kDa apoptosis-related cleavage fragment. The 85-kDa proteolytic PARP fragment was readily detected in cell lysates of cisplatin-treated NT2, NCCIT and S2 cultures, but not in the cell lysate of drug-treated 2102 EP cells (Table 1). Figure 2A shows a representative Western blot to illustrate the presence and absence of the 85-kDa PARP fragment in cisplatin-treated NCCIT cells and 2102 EP cells respectively. The apparent absence of proteolytic cleavage of PARP in cisplatin-treated 2102 EP cells confirmed the nuclear morphology data. These data on induction of apoptosis by cisplatin are in agreement with the MTT $IC_{90}$ values. The cell lines NT2 and NCCIT, with the highest sensitivity for cisplatin in the MTT assay [NT2, $IC_{90}$ 8.5 ± 4.4 μM (mean ± s.d.); NCCIT, 7.0 ± 0.3 μM], are the most sensitive for induction of apoptosis. The S2 cell line ($IC_{90}$ 15.2 ± 3.0 μM) takes an intermediate position, while in the resistant 2102 EP cell line ($IC_{90}$ 27.2 ± 3.6 μM) hardly any apoptosis could be detected.

As cisplatin resistance has been associated with reduced intracellular accumulation, we quantitated the total intracellular Pt levels of these TGCT cell lines immediately after cisplatin incubation (100 μM, 2 h). As shown in Table 1, NT2 and 2102 EP cells accumulated about equal amounts of Pt, while these cell lines showed a 3.2-fold difference in cisplatin sensitivity (MTT data). Notably, the differences in accumulation were completely paralleled by similar differences in Pt-DNA adduct formation (Table 1). The observed intracellular Pt levels and DNA platination in chemosensitive NT2 and chemoresistant 2102 EP cells excluded the possibility that increased efflux as a result of functional overexpression of an ATP-dependent glutathione *S*-conjugate pump (Ishikawa et al, 1994) accounted for the differential chemosensitivity. Hill et al (1994) suggested that the hypersensitivity of TGCTs might be related to a defective capacity to remove (repair) Pt-DNA adducts. However, chemosensitive NT2 and chemoresistant 2102 EP cells both removed about 40% of the Pt lesions from their genome within 8 h (data not shown), which indicates that the capacity to remove Pt/DNA adducts is identical and not impaired in these cell lines.

### Expression of p53, Bax and Bcl-2

We determined both the *p53* gene status by sequence analysis of the complete *p53* cDNA and the p53 response upon cisplatin treatment to evaluate the role of p53 in drug-induced apoptosis in our panel of TGCT cell lines. The sequence data revealed that NT2, S2 and 2102 EP cells are homozygous for wt *p53*, whereas NCCIT cells are hemizygous for *p53* containing one mutated allele carrying a 1-bp (G) deletion at position 949 of codon 272 (Table 1). The mutated *p53* allele with a stop codon at position 1114 encodes a truncated p53 protein of 347 amino acids that migrates faster than wt p53 protein (Figure 2B). This truncated p53 protein lacks the carboxy terminus encompassing the tetramerization, nuclear localization and DNA damage recognition site (Harris, 1996). The carboxy terminus of the p53 protein has been implicated in the induction of apoptosis (Wang et al, 1996), but nevertheless we clearly demonstrated that mt p53 NCCIT and wt p53 NT2 cells are equally sensitive to drug-induced apoptosis. Although no basal level of p53 protein was detected in S2 cells (Figure 2B), despite the presence of *p53* mRNA, drug-induced apoptosis was evident (Figure 1). NT2 and 2102 EP cells express a similar basal level of wt p53 protein, which was readily up-regulated upon cisplatin treatment. In contrast, NCCIT and S2 cells showed no cisplatin-induced p53 up-regulation. Consistent with cisplatin-induced wt p53 up-regulation, only wt p53 protein of NT2 and 2102 EP cells was capable to transactivate the *p21/WAF/CIP* gene upon DNA damage (El-Deiry et al, 1993), whereas mt p53-containing NCCIT cells and p53-non-expressing S2 cells could not transactivate this p53-responsive gene (Table 1). Thus, NCCIT and S2 cells are apparently p53 transactivation deficient, suggesting that there is no clear correlation between transactivation-competent p53 and induction of apoptosis in these TGCT cell lines. Although our results show that the presence of functional wt p53 is not required for cisplatin-induced apoptosis in some TGCT cell lines (NCCIT and S2), it still can not be excluded that wt p53 plays a role in the hypersensitivity of NT2 cells to cisplatin. Studies on inactivation of p53 function by introduction of the human papilloma virus E6 protein may serve to further define the role of p53 in TGCT cell lines. Our results concur with

© Cancer Research Campaign 1998

those of Cresta et al (1996), who concluded that the hypersensitivity of TGCT cell lines to etoposide-induced apoptosis was associated with functional p53. However, it should be noted that all the chemosensitive TGCT cell lines that they compared with chemoresistant bladder cancer cell lines contained wt p53, and in the study presented here we compared TGCT cell lines that differ in their p53 status.

The Bcl-2/Bax ratio is often an important determinant of apoptosis and determines the extent to which apoptosis is induced or suppressed (Oltvai and Korsmeyer, 1994). Our immunoblotting data, as presented in Figure 2B, reveal no correlation between endogenous Bcl-2 and Bax protein levels and susceptibility to drug-induced apoptosis. The endogenous levels of Bax protein in our panel of TGCT cell lines were found to be similar. No Bcl-2 protein expression was found in NCCIT cells, whereas readily detectable and similar Bcl-2 protein levels were found in the other TGCT cell lines. In addition to the endogenous Bcl-2 and Bax expression, which are inherent to the cell, we examined the effect of different doses of cisplatin (3.1–12.5 μM) on the expression of these death-modulating genes, which was monitored at different time points after treatment (6–72 h). Notably, Bcl-2 and Bax expression was not affected by cisplatin, irrespective of the dose and time point studied (Figure 2B). With respect to Bax expression as detected by Bax PAb, it should be noted that we found identical results with a Bax MAb that became available only recently (data not shown). Although no Bax up-regulation was apparent by immunoblotting, we have demonstrated previously heterogeneous Bax expression and up-regulation within the apoptotic NT2 population using flow cytometry (Boersma et al, 1997). However, no Bax up-regulation could be detected by flow cytometry in the other TGCT cell lines (AWM Boersma, personal communication). Our results indicate that the Bcl-2/Bax rheostat is probably not involved in cisplatin-induced apoptosis of TGCT cell lines.

In summary, our data suggest that, at least in our panel of TGCT cell lines, there seems to be no clear relationship between cisplatin-induced apoptosis and (a) growth characteristics, (b) cellular Pt accumulation, (c) DNA platination, (d) Pt-DNA adduct removal, (e) p53 status, (f) intrinsic Bcl-2 and Bax expression and (g) cisplatin-induced modulation of the Bcl-2/Bax ratio. The observed lack of correlation between p53 status and apoptosis induction may indicate that the presence of wt p53 is not absolutely required for the successful treatment of TGCTs. Further unraveling of the apoptotic pathway and identification of key determinants of chemosensitivity in TGCTs may lead to more appropriate and successful anti-cancer treatment modalities for tumour types that are now considered to be drug resistant.

### ABBREVIATIONS

Pt, platinum; FCM, flow cytometry; FITC, fluorescein isothiocyanate; $IC_{90}$, inhibitory concentration (90%); MTT, 3-[4,5-dimethylthiazol-2-yl]-2,5 diphenyltetrazoliumbromide; PI, propidium iodide; TGCT, testicular germ cell tumour; PARP, poly(ADP-ribose) polymerase

### ACKNOWLEDGEMENTS

We thank Dr B Vogelstein (The Johns Hopkins University School of Medicine, Baltimore) for providing the pCEP-WAF-1 plasmid. We also thank Erik CE Brouwer for assistance with Pt determinations by AAS and Rolph HAM Vossen (Laboratory of Anthropogenetics, Sylvius Laboratories, Leiden University, Leiden, The Netherlands) for his help with p53 sequence determinations. This work was supported in part by Grant DDHK 94-846 from the Dutch Cancer Society.

### REFERENCES

Boersma AWM, Nooter K, Burger H, Kortland CJ and Stoter G (1997) Bax upregulation is an early event in cisplatin-induced apoptosis in human testicular germ cell tumour cell line NT2, as quantitated by flow cytometry. *Cytometry* **27**: 275–282

Carmichael J, De Graff WG, Gadzar AF, Minna JD and Mitchell JB (1987) Evaluation of a tetrazolium-based semi-automated colorimetric assay: assessment of chemosensitivity testing. *Cancer Res* **47**: 936–947

Clarke AR, Purdie CA, Harrison DJ, Morris RG, Bird CC, Hooper ML and Wyllie AH (1993) Thymocyte apoptosis induced by p53-dependent and independent pathways. *Nature* **362**: 849–852

Cresta CM, Masters JRW and Hickman JA (1996) Hypersensitivity of human testicular tumours to etoposide-induced apoptosis is associated with functional p53 and a high Bax:Bcl-2 ratio. *Cancer Res* **56**: 1834–1841

Damjanov I, Horvat B and Gibas Z (1993) Retinoic acid-induced differentiation of the developmentally pluripotent human germ cell tumour-derived cell line, NCCIT. *Lab Invest* **68**: 220–232

Dive C and Hickman JA (1991) Drug–target interactions: only the first step in the commitment to a programmed cell death? *Br J Cancer* **64**: 192–196

Einhorn LH (1990) Treatment of testicular cancer: a new and improved model. *J Clin Oncol* **8**: 1777–1781

El-Deiry WS, Tokino T, Velculescu VE, Levy DB, Parsons R, Trent JM, Lin D, Mercer E, Kinzler KW and Vogelstein B (1993) WAF1, a potent mediator of p53 tumour suppression. *Cell* **75**: 817–825

Fan S, El-Deiry WS, Bae I, Freeman J, Jondle D, Bhatia K, Fornace Jr AJ, Magrath I, Kohn KW and O'Connor PM (1994) p53 Gene mutations are associated with decreased sensitivity of human lymphoma cells to DNA damaging agents. *Cancer Res* **54**: 5824–5830

Fisher DE (1994) Apoptosis in cancer therapy: crossing the threshold. *Cell* **78**: 539–542

Harris CC (1996) Structure and function of the p53 tumour suppressor gene: clues for rational cancer therapeutic strategies. *J Natl Cancer Inst* **88**: 1442–1452

Hill BT, Scanlon KJ, Hansson J, Harstrick A, Pera M, Fichtinger-Schepman AMJ and Shellard SA (1994) Deficient repair of cisplatin-DNA adducts identified in human testicular teratoma cell lines established from tumours from untreated patients. *Eur J Cancer* **30A**: 832–837

Huddart RA, Titley J, Robertson D, Williams GT, Horwich A and Cooper CS (1995) Programmed cell death in response to chematherapeutic agents in human germ cell tumour lines. *Eur J Cancer* **31A**: 739–746

Ishikawa T, Wright CD and Ishizuka H (1994) GS-X pump is functionally overexpressed in cis-diamminedichloroplatinum(II)-resistant human leukemia HL-60 cells and down-regulated by cell differentiation. *J Biol Chem* **269**: 29085–29093

Kaufmann SC, Desnoyers S, Ottaviano Y, Davidson NE and Poirier GG (1993) Specific proteolytic cleavage of poly(ADP-ribose polymerase: an early marker of chemotherapy-induced apoptosis. *Cancer Res* **53**: 3976–3985

Kerr JFR, Winterford CM and Harmon BV (1994) Apoptosis: its significance in cancer and cancer chemotherapy. *Cancer* **73**: 2013–2026

Korsmeyer SJ (1992) Bcl-2 initiates a new category of oncogenes: regulators of cell death. *Blood* **80**: 879–886

Lotem J and Sachs L (1993) Regulation by Bcl-2, c-myc, and p53 of susceptibility to induced apoptosis by heat-schock and cancer chemotherapy compounds in differentiation competent and defective myeloid leukemia. *Cell Growth Diff* **4**: 41–47

Lowe SW, Ruley HE, Jacks T and Housman DE (1993) p53-Dependent apoptosis modulates the cytotoxicity of anticancer agents. *Cell* **74**: 957–967

Masters JRW, Osborne EJ, Walker MC and Parris CN (1993) Hypersensitivity of human testis tumour cell lines to chemotherapeutic drugs. *Int J Cancer* **53**: 340–346

Miller SA, Dykes DD and Polesky HF (1988) A simple salting out procedure for extracting DNA from human nucleated cells. *Nucleic Acids Res* **16**: 1215

Miyashita T, Krajewski S, Krajewski M, Wang HG, Lin HK, Liebermann DA, Hofmann B and Reed JC (1994) Tumour suppressor p53 is a regulator of bcl-2 and bax gene expression in vitro and in vivo. *Oncogene* **9**: 1799–1805

© Cancer Research Campaign 1998

Oltvai ZN and Korsmeyer SJ (1994) Checkpoints of duelling dimers foil death wishes. *Cell* **79**: 189–192

Peng HQ, Hogg D, Malkin D, Bailey D, Gallie BL, Bulbul M, Jewett M, Buchanan J and Gross PE (1993) Mutations of the p53 gene do not occur in testis cancer. *Cancer Res* **53**: 3574–3578

Reed JC, Miyashita T, Takayama S, Wang H-G, Sato T, Krajewski S, Aimé-Sempé C, Bodrug S, Kitada S and Hanada M (1996) Bcl-2 family proteins: regulators of cell death involved in the pathogenesis of cancer and resistance therapy. *J Cell Biochem* **60**: 23–32

Sark MWJ, Timmer-Bosscha H, Meijer C, Uges DRA, Sluiter WJ, Peters WHM, Mulder NH and de Vries EGE (1995) Cellular basis for differential sensitivity to cisplatin in human germ cell tumour and colon carcinoma cell lines. *Br J Cancer* **71**: 684–690

Wang N, Perkins-Kl, Bronson DI and Fraley EE (1981) Cytogenetic evidence for premeiotic transformation of human testicular cancers. *Cancer Res* **41**: 2135–2140

Wang XW, Vermeulen W, Coursen JD, Gibson M, Lupold SE, Forrester K, Xu G, Elmore L, Yeh H, Hoeijmakers JHJ and Harris CC (1996) The XPB and XPD helicases are components of the p53-mediated apoptosis pathway. *Genes Dev* **10**: 1219–1232

Zakut-Houri R, Bienz-Tadmor B, Givol D and Ohren M (1985) Human p53 cellular tumour antigen: cDNA sequence and expression in COS cells. EMBO J **4**: 1251–1255

Zhan Q, Fan S, Bae I, Guillouf C, Liebermann DA, O'Connor PM and Fornace AJ (1994) Induction of bax by genotoxic stress in human cells correlates with normal p53 status and apoptosis. *Oncogene* **9**: 3743–3751

© Cancer Research Campaign 1998